# Towards a more integrated and gender-sensitive care delivery for victims of sexual assault: key findings and recommendations from the Belgian sexual assault care centre feasibility study

**DOI:** 10.1186/s12939-018-0864-3

**Published:** 2018-09-24

**Authors:** Bavo Hendriks, Anke Marie-Josée Aimé Vandenberghe, Laura Peeters, Kristien Roelens, Ines Keygnaert

**Affiliations:** 1 International Centre for Reproductive Health (ICRH), Department of Uro-Gynaecology, Faculty of Medicine and Health Sciences, Ghent University, Corneel Heymanslaan 10, UZP114, B-9000 Ghent, Belgium; 2 Department of Obstetrics and Gynaecology, Faculty of Medicine and Health Sciences, Ghent University, Ghent University Hospital, Corneel Heymanslaan 10, UZP3, B-9000 Ghent, Belgium

**Keywords:** Sexual assault, Sexual violence, Rape, Sexual and gender minorities, Gender-sensitive care, Integrated care, Sexual assault Centre

## Abstract

**Background:**

Sexual assault remains a major threat to public health, affecting every gender, gender identity and sexual orientation. Following the Belgian ratification of the Istanbul Convention in 2016, the feasibility of a Belgian sexual assault centre model was investigated, aiming to provide more integrated and patient-centred health and judiciary services to victims of sexual assault. By actively involving health professionals, police and judiciary system representatives, as well as victims themselves, this feasibility study eventually fed into the Belgian Sexual Assault Care Centre model. In this process, this paper assessed current Belgian health services and the degree to which the implementation of this model could contribute to both a more integrated and gender-sensitive care delivery. Findings from this study and the subsequent recommendations aim to contribute to similar reforms in other countries that have already taken or are about to take steps towards an integrated, multi-agency support framework for victims of sexual assault.

**Methods:**

A qualitative, descriptive analysis of the survey response of 60 key health professionals (*N* = 60) representing 15 major Belgian hospitals was first conducted. Comparing their approach with the international guidelines and standards, a Strengths Weaknesses Opportunities and Threats analysis of the current sexual assault health services and their potential transition to the Sexual Assault Care Centre model was then executed.

**Results:**

Despite adequate equipment, the clear fragmentation of health services and limited follow-up hamper an integrated care delivery in most hospitals. Only three hospitals differentiated their sexual assault care protocol based on the victim’s gender, gender identity and sexual orientation. A striking unawareness among health professionals of sexual assault in male victims, as well as in gender and sexual minorities further hampers a gender-sensitive care delivery.

**Conclusions:**

The Sexual Assault Care Centre model aims to counter most of the current sexual assault health services’ weaknesses and threats hampering an integrated care for victims of sexual assault. Further research and training of health professionals are however required in order to tune this integrated form of care to sexuality and gender-based differences in victims’ already multi-faceted healthcare needs.

## Background

### Definitions of sexual assault

The World Health Organisation defines sexual assault or sexual violence as ‘any sexual act that is perpetrated against someone’s will, committed by any person regardless of their relationship to the victim, in any setting. It includes but is not limited to rape, attempted rape and sexual slavery, as well as unwanted touching, threatened sexual violence and verbal sexual harassment’ [1]. Definitions of sexual assault can however show clear international and interdisciplinary variations, with sometimes important consequences for both victims and perpetrators. The Belgian penal code for example only distinguishes between indecent assault and rape. By considering rape as ‘any act of sexual penetration of any kind and by any means whatsoever committed to a person without consent’ [2, 3], the Belgian law emphasises the penetration of the victim. Incidents where the victim was coerced to penetrate the perpetrator or a third person vaginally, anally or orally are therefore considered as indecent assault, resulting in a less severe penalty for the perpetrator [3].

### Prevalence rates of sexual assault

Due to varying definitions, study setup, as well as differing sociocultural contexts, prevalence rates of sexual assault also tend to vary globally among different studies. According to the World Health Organisation, sexual assault can nevertheless be considered as a global problem, ‘neither rare nor unique to any particular region in the world’ [4].

Recent epidemiologic research, conducted in the Flemish region of Belgium, estimates that 22.3% of females under 18 years old and 13.8% of females older than 18 years are exposed to sexual assault. Prevalence rates in the male population vary from 10.7% in males under 18 years old to 2.4% in males older than 18 years [5]. The Flemish prevalence of sexual assault in people identifying as lesbian, gay, bisexual and/or transgender rises to 41.1%, while particularly 31.7% of transgender people report to have been sexually assaulted at least once in their lives. Furthermore, these prevalence rates appear to be significantly higher in non-heterosexual men than in non-heterosexual women [6].

A study conducted in 10 European countries, including Belgium, showed victimisation in 19.7 to 52.2% of young women, aged between 18 and 27 years, and in 10.1 to 55.8% of young men between 18 and 27 years old [7]. According to a European Union survey, 26% of lesbian, gay, bisexual and/or transgender people reported to have experienced physical or sexual aggression in the past 5 years, while this counted for 34% of transgender respondents solely [8].

### Health consequences

The main physical consequences of sexual assault consist of acute anogenital and other physical injuries, sexually transmitted infections, as well as unwanted pregnancy, dyspareunia and other sexual and reproductive complications in biologically female victims. The risk of anal injuries appears to be relatively higher in male than in female victims, while injuries to other body parts are more frequent in female victims [9, 10]. Furthermore, psychosomatic disorders, such as irritable bowel syndrome and chronic pain syndrome, as well as risky and unhealthy behaviour patterns, both sexually (e.g. unprotected sex, sex under the influence of alcohol or drugs, having multiple and/or high-risk sexual partners) and non-sexually (e.g. eating disorders, smoking, alcohol and drug abuse), are commonly reported in the long term in both male and female victims [9, 11].

The psychological response to sexual assault can vary greatly among victims based on their perception of the circumstances and their relationship with the perpetrator. In most cases feelings of shock, shame, fear, confusion, guilt and stress peak within 3 weeks post assault and can linger on for 1 to 2 months, often resulting in anxiety and depression. Post-traumatic stress disorder can further develop in 30 to 94% of victims, with a significant risk of re-traumatisation when insensitively addressed and/or insufficiently supported during disclosure and further care delivery [12].

The conflict of male victimisation with the stereotypical perception of men being the sex-dominant gender is considered as an important sociocultural barrier preventing disclosure in male victims [13]. Moreover, a higher incidence of internalised homophobia in men together with the taint of homosexuality on male-on-male rape can also worsen feelings of shame, confusion and guilt within the first weeks post assault [14, 15]. This sociocultural context combined with the possible physiological response in men of erection and ejaculation provoked by high levels of anxiety or anal stimulation can further add up to the fear of prejudice and disbelief during disclosure [13–18]. These barriers to disclosure are therefore generally believed to increase the risk of psychological symptoms, post-traumatic stress disorder and other trauma-related disorders in male compared to female victims [13].

### Gender-sensitive care

A growing body of evidence suggests an important role for gender stereotypes and gender inequality in the sociocultural context wherein sexual assault can arise, as well as in the occurrence, circumstances, risk factors and health consequences of sexual assault [7, 9–21]. This gendered aspect of sexual assault can therefore lead to significant differences in victims’ already multi-faceted healthcare needs based on their gender, gender identity and sexual orientation [9, 22–26]. However, with health equity for all being considered as a fundamental human right and once again embodied in the 2030 United Nations’ Sustainable Development Goals [27, 28], most international guidelines on the care delivery for male victims of sexual assault still remain medically focused, contradictory and concise, while sexual and gender minorities continue to be generally overlooked [1, 13, 22–25, 29–33].

### Integrated care

On the contrary, the importance of an integrated and coordinated health and judiciary service provision to victims of sexual assault is unanimously acknowledged by the World Health Organisation, as well as by other international organisations, guidelines and standards [1, 29–31]. This approach however requires a well-coordinated collaboration of a variety of professions and institutions, ranging from health professionals and social service providers, forensic medicine, police services, the judiciary system, as well as the involvement of victims themselves, in order to provide more timely, accessible and efficient sexual assault services [1].

In this context, the sexual assault centre model often serves as a guiding example. However internationally varying in title and setup, the common thread of these sexual assault centres generally remains a hospital-based health facility that integrates medical, psychosocial and forensic acute and follow-up care, accessible 24 h a day, 7 days a week, in the context of a clearly defined referral and multi-agency network, including but not limited to police and judiciary services [1, 29, 34].

To date, 33 Council of Europe member states have ratified the Convention of Istanbul and herewith committed themselves to develop this kind of integrated and multi-agency support framework, most suited to their individual health and judiciary system [35]. After Belgium joined in 2016, the Belgian Federal Secretary of State of Equal Opportunities commissioned to investigate the feasibility of a Belgian sexual assault centre model, actively involving health professionals, police and judiciary system representatives, as well as victims themselves [36, 37]. The resulting Sexual Assault Care Centre model, inspired by the leading examples of Ireland, the United Kingdom, Norway, Denmark, the Netherlands and the United States, was consecutively piloted in 2017–2018 in the Belgian cities of Ghent, Brussels and Liege [38–46].

### The Belgian Sexual Assault Care Centre model in an international context

The Sexual Assault Care Centre model intends to guarantee a 24/7 accessibility to medical, psychosocial, and forensic care delivered by a Sexual Assault Nurse Examiner, while being supported by a multidisciplinary Sexual Assault Referral Team, comparable to the Irish, Danish, Norwegian, Dutch and American model [38, 40–46]. Out of respect for the victim’s privacy, each centre would be discretely accessible through an alternative entrance [46]. Whereas nurses are only entitled to an assisting role in the European models as mentioned above, the Sexual Assault Nurse Examiner would be fully responsible for the first psychosocial and acute medical care, as well as the forensic examination, which is also the case in the United States [38, 40–48]. Whether or not the victim has decided to report the assault to the police, evidence could still be collected and preserved for a possible subsequent judiciary procedure, as is done in the Irish Sexual Assault Treatment Units [38, 46].

Considering the short- and long-term physical health consequences of sexual assault, the World Health Organisation recommends medical follow-up visits at 2 weeks, 3 and 5 months post assault [9, 10, 29]. As for the psychological follow-up of the victim, it is recommended to apply watchful waiting during the first one to 3 months [12, 30]. Similar to the Dutch Sexual Violence Centres, the Sexual Assault Care Centre model aims to assign a psychosocial case manager to contact the victim at least four times in the first month post assault, in order to coordinate the provided psychosocial and medical follow-up and referrals [42, 46]. The victim would also be offered two free consultations with a trauma psychologist at the care centre, as well as at the AIDS Referral Centre of the concerning hospital, both in the first week and 1 month post assault [46]. In case the victim develops signs of post-traumatic stress disorder, up to 20 free psychological counselling sessions could subsequently be provided for several months. A follow-up consultation with a gynaecologist, urologist, paediatrician or geriatrician could additionally be offered after one month. For other assistance, the Sexual Assault Care Centre focus would remain on the referral of the victim [46].

As further required by the Istanbul convention and in accordance with international guidelines and standards, all health professionals and police officers connected to the Sexual Assault Care Centre must follow a common training on sexual assault phenomenology and gender-sensitive sexual assault service provision. The Sexual Assault Nurse Examiners, case managers and trauma psychologists are subsequently required to attend an additional function-specific training [34–46].

### Aims and relevance

Sexual assault remains a major threat to public health due to its intrusive physical and mental health consequences for victims, their families and confidants both on the short and long term. The gendered, sociocultural context wherein sexual assault can arise however tends to differentiate victims’ already multi-faceted healthcare needs based on their gender, gender identity and sexual orientation. As part of the Belgian Sexual Assault Care Centre feasibility study, this paper’s aims are therefore twofold:

- To assess current Belgian health services in the context of international guidelines and standards.
- To evaluate the degree to which the implementation of the Belgian Sexual Assault Care Centre model could contribute to both a more integrated and gender-sensitive care delivery for victims of sexual assault.

The resulting recommendations consequently aspire to contribute to a more equitable and inclusive approach to sexual assault in countries across the globe that have already taken or are about to take steps towards an integrated, multi-agency sexual assault support framework.

## Methods

In order to assess current sexual assault health services in potential Belgian Sexual Assault Care Centre hospitals, a survey was first conducted. With adequate HIV post-exposure prophylaxis and follow-up care being an essential part of an integrated sexual assault care delivery [1, 29, 30, 33, 34], only hospitals serving as an AIDS Referral Centre were eligible for inclusion in the Belgian Sexual Assault Care Centre feasibility study. Seventeen Belgian hospitals matched this inclusion criterion. Subsequently, the respective medical and nursing coordinators of each hospital were invited by the Belgian federal Secretary of State of Equal Opportunities to participate. For every hospital, the health professionals most involved in providing sexual assault health services in the departments of emergency medicine, gynaecology, urology, psychiatry, paediatrics, the AIDS Referral Centre and the social service were identified and invited to complete a printed survey. Depending on the participant’s preference, the survey could also be completed in a structured face-to-face or phone interview. Along with the survey, an informed consent form and information letter was sent, providing more details on the study, the possible risks and benefits of participation, and the implemented privacy policy. Only the health professionals, from whom a signed consent form and completed survey was obtained, were included in our study sample.

Eventually, 60 of the 159 invited key health professionals (*n* = 60), coming from 15 of the 17 included Belgian hospitals (*N* = 15), completed the survey. Eight of these 60 health professionals preferred a structured face-to-face interview. The remaining 52 health professionals chose to complete a printed survey. No one opted for a phone interview.

The survey predominantly consisted of closed, semi-open and a few open questions. Topics covered were the respondent’s profile, the respondent’s knowledge of sexual assault, their attitudes and practices in sexual assault care delivery, the current sexual assault health services provided in the respondent’s hospital, as well as the respondent’s appreciation of these current health services and the Sexual Assault Care Centre model. A digital dataset was then constructed using IBM SPSS Statistics 23. The different responses to the survey’s open questions were coded and compared, before being categorised in different values and integrated into the dataset, along with the responses to the closed and semi-open questions.

For this paper, a qualitative, descriptive analysis of the respondents’ profile, the respondents’ knowledge of sexual assault, their attitudes and practices in sexual assault care delivery, the current sexual assault health services provided in the respondents’ hospitals, as well as the respondents’ appreciation of the Sexual Assault Care Centre model was then conducted. The current sexual assault health services of each hospital were analysed based on the dominant response of the respective respondents of that hospital. The respective hospital’s response to a question was classified as ‘unclear’ when no dominant response group could be identified.

Finally, comparing the results of the descriptive data analysis to the international guidelines and standards [1, 9, 10, 12, 13, 29–34, 38–45, 48], a Strengths Weaknesses Opportunities and Threats analysis of the current sexual assault health services and their potential transition to the Sexual Assault Care Centre model was executed. Based on the emerging strengths, weaknesses, opportunities and threats, the degree to which the implementation of the Belgian Sexual Assault Care Centre model could contribute to both a more integrated and gender-sensitive care delivery for victims of sexual assault will be evaluated in the discussion section of this paper.

## Results

### Respondents’ profile

Table 1 presents the principal demographics of the 60 key health professionals included in our sample.

**Table 1:** Respondents’ profile

	Count (n)	Percentage (%)
Gender (*N* = 60)		
Male	22	37
Female	38	63
Transgender	–	–
Age (*N* = 60)		
25–29 years	1	2
30–39 years	15	25
> 40 years	44	73
Work experience in years (*N* = 58)		
< 2 years	5	9
2–10 years	20	34
11–20 years	20	34
> 20 years	13	22
Departments (*N* = 60)		
Emergency medicine	10	17
Gynaecology	11	18
Urology	5	8
Psychiatry	5	8
Paediatrics	11	18
AIDS Referral Centre	10	17
Social service	7	12
Forensic medicine	1	2

### Knowledge of sexual assault and attitudes and practices in sexual assault care delivery

Every respondent (*n* = 60; 100%) agreed that sexual assault is a major threat to global health. The majority (*n* = 55; 91%) believed this is also the case for public health in Belgium.

Based on the prevalence rates derived from recent research, the respondents were subsequently asked about their estimates of the prevalence of sexual assault in Belgium [5, 6]. The majority of respondents (*n* = 34; 58%) agreed to the given prevalence rate of 15% in females older than 18 years, while a dominant 43% (*n* = 26) were not familiar with the prevalence rate in the female population under 18 years old. Furthermore, the majority had no idea about the prevalence rates of sexual assault in the male population under 18 years old (*n* = 35, 58%), males older than 18 years (*n* = 33; 55%), nor in lesbian, gay, bisexual (*n* = 37; 62%) and transgender people (*n* = 35; 58%).

Most respondents indicated being sufficiently skilled (*n* = 44; *N* = 57) and experienced (*n* = 41; *N* = 57) in discussing sexual assault with their patients. However, one third (*n* = 20; *N* = 56) reported to encounter practical problems in discussing sexual assault due to the current practical organisation of their department. In this context, almost all respondents agreed that the new Sexual Assault Care Centre model would provide a more focused (*n* = 55; *N* = 58), multidisciplinary framework (*n* = 57; *N* = 58) to work in, with less pressure of other patients waiting (*n* = 49; *N* = 58). Most respondents subsequently agreed that the Sexual Assault Care Centres would be able to provide both more timely (*n* = 44; *N* = 58) and specialised health services (*n* = 57; *N* = 58), as well as long-term follow-up (*n* = 52; *N* = 57) for victims of sexual assault [36].

### Sexual assault care protocols

Thirteen of the 15 included hospitals had already developed and implemented a standardised protocol on the provision of sexual assault health services (*N* = 13). Three of these 13 hospitals explicitly differentiated their protocols based on the victim’s gender, gender identity and sexual orientation.

### Accessibility of care

Every hospital (*n* = 15; *N* = 15) guarantees a 24/7 accessibility of the emergency ward, while in the social service this would only be the case in six of them.

Only one hospital offers integrated care for victims in its emergency ward exclusively. In the other 14 hospitals, care is offered in different departments, such as the departments of gynaecology, paediatrics and emergency medicine. In every hospital however, the emergency ward is involved in the care for victims of sexual assault.

### Infrastructure, equipment, tests and treatments

Table 2 illustrates the minimal required infrastructure and disposal of equipment in the 15 included hospitals, as well as their ability to provide the minimum of tests and treatments required in the provision of sexual assault health services according to the World Health Organisation. The items marked with an asterisk are considered as essential [29]. Fewer than half of the respondents (*n* = 22; *N* = 60) answered the questions on the equipment of their hospitals.

**Table 2:** Infrastructure, equipment, tests and treatments in included hospitals, *N* = 15

	Disposable (n)	Not disposable (n)	No idea (n)	Unclear (n)
Equipment				
Thermally neutral examination room	15	–	–	–
Examination couch	15	–	–	–
Mobile light source	15	–	–	–
Colposcope	15	–	–	–
Sexual Agression Set	15	–	–	–
Camera	15	–	–	–
Shower	14	1	–	–
Hand basin	15	–	–	–
Toilet	15	–	–	–
Towels	15	–	–	–
Sanitary items (e.g. pads, cloths)	15	–	–	–
Clothing	14	1	–	–
Telephone	15	–	–	–
Consent form	14	1	–	–
Tests				
Pregnancy tests	14	–	–	1
Chlamydia Trachomatis	14	–	–	1
Neisseria Gonorrhea	14	–	–	1
Bacterial vaginosis	14	–	–	1
Syphilis	14	–	–	1
HIV	14	–	–	1
Repeated HIV test after 6 weeks, 3 and 6 months	14	–	–	1
Treatments				
Emergency contraception	14		1	–
Tetanus prophylaxis	12	3	1	–
Hepatitis B prophylaxis	13	1	1	–
HIV post-exposure prophylaxis	13	1	1	–

### Forensic care

The respective single respondents of two of the 15 included hospitals indicated each time that forensic care did not belong to their domain of expertise, while the majority of respondents of a third hospital had no idea on the forensic care delivery in their hospital. The results illustrated by Table 3 therefore apply to the remaining twelve hospitals (*N* = 12). A Sexual Aggression Set is a pre-packaged set containing all items required for the collection of forensic evidence exclusively in victims of sexual assault [44, 45]. According to current Belgian law, the usage of these sets is still reserved for physicians exclusively, while nurses are being restricted to an assisting role [45].

**Table 3:** Sexual Aggression Set procedure and responsible forensic examiner in included hospitals, *N* = 12

	Always/sometimes (n)	Rarely/never (n)	No idea (n)	Unclear (n)
Forensic examiner in adult victims				
Obstetrician/gynaecologist	10	–	1	1
Urologist	3	4	1	4
Emergency physician	1	8	1	2
Forensic pathologist	1	8	1	2
Nurse	–	11	1	–
Forensic examiner in minor victims				
Obstetrician/gynaecologist	7	–	–	5
Urologist	–	3	–	9
Emergency physician	1	6	–	5
Forensic pathologist	3	2	1	6
Paediatrician	5	2	–	5
Nurse	–	10	–	2

### Follow-up

Table 4 illustrates the time of follow-up of the victim in the concerning hospitals where follow-up at the social service (*N* = 10) and the department of gynaecology/urology (*N* = 9) is offered. Follow-up at the AIDS Referral Centre is offered in seven of the included hospitals (*n* = 7; *N* = 15).

**Table 4:** Time of follow-up at the social service (*N* = 10) and gynaecology/urology department (*N* = 9)

	In hospital (n)	Other (e.g. telephone) (n)	Depends on victim/situation (n)	No follow-up (n)	No idea (n)	Unclear (n)
Social service						
3 days	3	–	5	2	–	–
2 weeks	1	–	1	5	1	2
1 month	2	–	1	5	1	1
3 months	1	–	1	6	1	1
6 months	1	–	1	6	1	1
Other	–	1	2	6	1	–
Gynaecology/urology						
3 days	3	–	2	4	–	–
2 weeks	3	–	1	4	–	1
1 month	1	–	–	6	–	2
3 months	–	–	1	7	–	1
6 months	–	–	–	7	–	2
Other	–	–	1	7	–	1

In five hospitals (*n* = 5; *N* = 15) the actual effectuation of the provided follow-up is ascertained.

### Training

Eight hospitals (*n* = 8; *N* = 15) offer training on the provision of sexual assault health services in general, while specialised training on a sensitive care delivery for lesbian, gay, bisexual and/or transgender victims is offered in one hospital. However, according to the majority of their representing respondents, eleven hospitals would offer adequate training in all departments involved in the provision of sexual assault health services.

## Discussion

### Strength: Hospitals’ equipment and ability to provide tests and treatments

Comparing our findings of current sexual assault health services provided in the included hospitals with international guidelines and standards, the main strength of current Belgian sexual assault health services might be the hospitals’ disposal of the required equipment and infrastructure, as well as their ability to provide the necessary tests and treatments [29]. This well-equipped healthcare setting could serve as an important advantage for the transition to the Sexual Assault Care Centre model.

### Weaknesses: Fragmented health services, limited follow-up and unawareness

The World Health Organisation strongly recommends sexual assault health services to integrate medical, psychosocial and forensic care [1, 29, 30]. However, health services provided by most of the included hospitals are generally scattered across different departments. Differences in accessibility of services are in their turn clearly illustrated by the emergency ward being accessible 24/7 in every hospital, while the social services often operate during office hours only. The number of respondents indicating that their hospital’s equipment or the questions on the Sexual Aggression Set procedure do not apply to their domain of expertise further contributes to the idea of a fragmented care. This clear fragmentation not only hampers integration and coordination of care. It can also require victims to disclose multiple times, increasing their risk of re-traumatisation [12, 29, 31]. By enrolling a Sexual Assault Nurse Examiner, specialised in providing acute medical, forensic and psychosocial care, while reducing the current physicians’ responsibility to a mere supporting role, the Sexual Assault Care Centre model aims to counter this lack of integration of health services [42–44]. The resulting task shift not only relieves physicians from the current practical problems experienced by one third of our study sample, but can also lead to a more thorough forensic examination, impacting the possible consecutive judiciary procedure and outcome [44, 46].

Considering the follow-up of victims of sexual assault at the social service as well as at the departments of gynaecology/urology of the included hospitals, it appears that follow-up is mostly limited to a short-term period of 2 weeks post assault, if provided. By appointing a case manager from day one after discharge, the Sexual Assault Care Centres intend to guarantee a more coordinated medical and psychosocial follow-up and referral of the victim, both on the short- and long-term. Our survey however made no clear distinction between follow-up at the gynaecology and the urology department. Due to the low representation rate of urologists in our sample, the involvement of urologists in the provided sexual assault health services can nonetheless be questioned, primarily hampering a gender-sensitive care delivery for biologically male victims.

Although most of the included hospitals have implemented a standardised protocol on the provision of sexual assault health services, only three of them committed themselves to tune their protocols based to the victim’s gender, gender identity and sexual orientation. This clear ignorance also manifests itself in the health professionals’ training, directly resulting in a lack of awareness on sexual assault in men, as well as in gender and sexual minorities. Yet, a basic theoretical and sociocultural understanding of sexual assault and its resulting gender- and sexuality-based differences in victim’s already multi-faceted health care needs is considered to help health professionals in creating a sense of safety and trust for victims. It could also facilitate the identification and overcoming of barriers in victims’ disclosure and further care process, resulting in a more effective relationship between victims and health professionals [24, 26]. Despite their lack of awareness on sexual assault in both men and gender and sexual minorities, most respondents indicate being sufficiently skilled and experienced in discussing sexual assault with their patients, as well as being adequately trained on the provision of sexual assault health services. The majority of health professionals in our sample thus seem to trust on their individual experience and skills, unaware of the fundamental importance of a theoretical and sociocultural understanding of sexual assault as a prerequisite for gender-sensitive care [22–25]. By offering an evidence-based training to all connected health professionals, the Sexual Assault Care Centre model intends to counter this lack of awareness in order to attain both a more standardised and gender-sensitive care delivery [1, 30, 31, 44].

### Opportunities: Growing national and international support and commitment

Together with a growing body of evidence on sexual assault health services and gender-sensitive care delivery [7, 9–21], increasing international media coverage and political commitments such as the Istanbul Convention create an excellent opportunity for sexual assault health services reforms to be developed and implemented. Moreover, reforms such as the Sexual Assault Care Centre model seem to be widely supported by health professionals involved in the field, as illustrated by our study findings.

### Threats: Stigma, politics and legislation

Sociocultural barriers and stigma continue to prevent victims from disclosure and seeking care, primarily threatening the accessibility of care [12–18]. The Sexual Assault Care Centre model therefore initially aims to secure and protect the victim’s privacy by providing a discrete and alternative entrance to each centre. Secondly, as mentioned above, all connected health professionals and police officers must follow a common training on sexual assault service provision and gender-sensitive care delivery, educating them on identifying and overcoming these sociocultural barriers in victims’ disclosure and further care process [44].

As long as the Sexual Assault Care Centre model is not structurally embedded in the Belgian judiciary and healthcare system, both national health policy reforms such as austerity measures and hospital-network management as well as possible future electoral shifts in the political landscape could further delay or hamper the implementation and expansion of Sexual Assault Care Centres [34, 44]. Despite several legislative reforms that have already been made in the developing process of the Sexual Assault Care Centre model, the current legal distinction between indecent assault and rape continues to primarily hamper a gender-sensitive judiciary approach to sexual assault in Belgium [3].

### Recommendations

Profiting from a well-equipped healthcare setting, as well as from increasing national and international support and commitment, the newly developed Sexual Assault Care Centre model aims to counter most of the current sexual assault health services’ weaknesses and threats hampering an integrated care. Recommendations can however be made for this model to further tune this form of integrated care to victims’ particular sexuality- and gender-based healthcare needs.

First, more research is required on the occurrence, circumstances, risk factors and mental and physical health consequences of sexual assault in male and especially lesbian, gay, bisexual and/or transgender victims. The resulting academic corpus can then be matched to the available evidence on sexual assault in female victims, so that similarities and differences in their particular healthcare needs become clear. In this process, involving organisations working with or representing victims of sexual assault and/or sexual and gender minorities should be of utmost importance.

Secondly, the resulting evidence can then be used to not only educate health professionals on general sexual assault phenomenology and sexual assault health services, but also on the gendered, sociocultural context wherein sexual assault can arise and its resulting gender- and sexuality-based differences in victims’ healthcare needs. Since victims can disclose on every level in the healthcare sector, the introduction of these educational trainings should be considered in all under- and postgraduate medical and paramedical curricula [19]. Moreover, by collaborating with universities and research centres, the Sexual Assault Care Centres could play a key role in running both national research and educational projects aiming to inform and sensitise public opinion on sexual assault phenomenology [34, 38].

Consecutively, a more extensive scientific basis, as well as a more profound understanding of sexual assault phenomenology among health professionals could serve as a catalyst for the development and implementation of more gender-sensitive care standards and guidelines, both on a national and international level.

### Limitations

Due to a low response ratio in some parts of the survey, as well as a possible voluntary or involuntary response bias, some findings in this paper require careful interpretation. Furthermore, since the great majority of respondents preferred to complete a printed survey, the motives behind their responses and non-responses could not be explored. Despite these traditional qualitative inquiry related restrictions, this paper does offer valuable insights into sexual assault health services provision, highlighting the importance of a gender-sensitive care delivery.

## Conclusion

Sexual assault remains a major threat to public health. The gendered, sociocultural context wherein sexual assault can arise however tends to differentiate victims’ already multi-faceted healthcare needs based on their gender, gender identity and sexual orientation. International organisations, guidelines and standards stress the importance of an integrated and coordinated service provision to victims of sexual assault, while guidelines on a gender-sensitive care delivery often remain medically focused, contradictory and concise. Aiming to assess current Belgian sexual assault health services, this paper showed a clear fragmentation of care, limited follow-up of victims and a striking unawareness among health professionals of the importance of a theoretical and sociocultural understanding of sexual assault as a prerequisite for a gender-sensitive care delivery. However, profiting from a well-equipped healthcare setting, as well as from increasing national and international support and commitment, the newly developed Belgian Sexual Assault Care Centre model intends to guarantee a more integrated care for victims of sexual assault. Further research and training of health professionals are however required in order to tune this integrated form of care to sexuality and gender-based differences in victims’ healthcare needs. These recommendations should not only apply to the Belgian Sexual Assault Care Centres, but to all other countries across the globe which already took or are about to take steps towards an integrated, multi-agency support framework for victims of sexual assault. By recognising and adequately addressing the different sexuality and gender-based healthcare needs of victims, comparable healthcare reforms could consecutively lead to a more inclusive and equitable approach of sexual assault, lowering the threshold for all victims to seek the highest attainable standard of care they need.
